# The Role of Collectivism–Individualism in Attitudes Toward Compliance and Psychological Responses During the COVID-19 Pandemic

**DOI:** 10.3389/fpsyg.2021.600826

**Published:** 2021-10-28

**Authors:** Wen S. Xiao

**Affiliations:** Teacher Education School, Shaoguan University, Shaoguan, China

**Keywords:** COVID-19, psychological responses, collectivism, individualism, attitudes toward compliance

## Abstract

This study examined the role of individual differences in horizontal and vertical individualism and collectivism, trust and worries, and concerns about COVID-19 in predicting the attitudes toward compliance of health advice and psychological responses during the COVID-19 pandemic. Chinese university students (*N*=384, 324 female) completed measures of individualism and collectivism, trust, attitudes toward compliance, and psychological responses to the pandemic. Results showed that not only vertical collectivist orientation but also horizontal individualist orientation significantly predicted higher willingness to comply, whereas vertical individualist orientation significantly predicted lower willingness to comply. Vertical individualist and vertical collectivist orientations predicted higher psychological response in terms of distress, anxiety, and depression, while horizontal collectivistic orientation significantly predicted less psychological problems. Implications of the effect of individual-level cultural orientations on attitudes toward public health compliance and psychological well-being during global health crises are discussed.

## Introduction

The coronavirus (COVID-19) pandemic posed a threat to people’s physical health, lifestyle, and psychological well-being. Evidence has shown that COVID-19 is highly contagious, capable of asymptomatic transmission, and causing death or series illness (Guan et al., 2020). Globally, nearly one-third of the world population was forced into lockdown (Kaplan et al., 2020). People’s social life had undergone enormous changes with forced isolation, movement restriction, and active government surveillance (Ullah and Khan, 2020). Although these measures effectively slowed the transmission of the virus (Cowling et al., 2020), adverse psychological impact and negative emotions appeared due to reduced autonomy and lack of real-life interactions (Kowal et al., 2020). However, in the absence of effective treatment, vaccines, or widespread population immunity, behaviors that can prevent COVID-19’s spread (e.g., wearing masks, staying home) only work effectively when practiced collectively. The aim of current study was to understand the critical predictors of people’s attitudes toward compliance of the health advice and negative psychological responses during the pandemic. Specifically, the role of individual-level cultural orientations of vertical and horizontal individualism and collectivism and trust was investigated.

## Health Compliance During 2020 Covid-19

To slow the spread of the virus, WHO advised the public to take some simple precautions, such as physical distancing, wearing a mask, keeping rooms well ventilated, avoiding crowds, cleaning hands, and coughing into a bent elbow or tissue. Governments around the world have responded differently to this pandemic by implementing the mitigating measures and thus achieved differential success (Baniamin et al., 2020). In some countries, for example, China and South Korea, public health officials have the authority to make these measures compulsory (Beech, 2020; Fisher and Sang-Hun, 2020). Chinese government imposed a complete lockdown of the city Wuhan with three weeks into the epidemic (Graham-Harrison and Kuo, 2020). Within days, the quarantine was extended to additional provinces and cities. More than 50 million people stayed at home and socially isolated themselves to prevent being infected, leading to a “desperate plea” (Horton, 2020). After an 11-week lockdown, Wuhan lifted restrictions on outgoing travel since the number of newly infected cases decreased dramatically. Korean’s successful experience resulted from a combination of testing and contact-tracing apps (Lee and Lee, 2020). But other countries, such as United Kingdom and the Netherlands, did not take any significant steps to combat the spread of the virus or if they took any strategies, that appeared as insufficient and eventually proved ineffective (Baniamin et al., 2020). The fact that different populations respond to epidemics differently suggests that managing crises like the COVID-19 need to consider people’s attitude, citizen trust, and culture (Van Bavel et al., 2020).

Wearing a face mask and social distancing are the most effective behavioral measures against infection and spread of the virus (Dehning et al., 2020; Feng et al., 2020; Leung et al., 2020). China was one of the few countries that responded to the epidemic outbreak swiftly by interventions including improved rates of diagnostic testing, clinical management, rapid isolation of suspected and confirmed cases and contacts, and most notably, restrictions on mobility (Kraemer et al., 2020). The combination of interventions implemented in China was clearly successful in mitigating spread and reducing local transmission of COVID-19 (Zhang et al., 2020a). Comparatively, Western countries experienced much more difficulties in managing the outbreak. Folk wisdom and anecdotal observation is that individualistic cultural values pose an obstacle to quelling COVID-19 transmission because measures, such as quarantining, social distancing, and even mask-wearing, are viewed as threats to individual freedom, self-reliance, and personal liberty (Webster et al., 2021). Thus, in democratic societies, following the social distancing advice is a function of citizen discretion and relies heavily on voluntary compliance (Tyler and Jackson, 2014). For the above-stated reasons, it is important to understand why people differ in their opinions about using the protective measures voluntarily.

## Psychological Responses Toward the Covid-19 Pandemic

From a psychological perspective, the COVID-19 pandemic and associated lockdown are characterized with uncertainty, ambiguity, and loss of control, which are known to trigger stress and emotional distress, anxiety, and depression (Reger et al., 2020). The potential COVID-19-related stressors, including worries about one’s own health and that of loved ones, economic disruption and loss, lifestyle disruptions, social isolation, and loneliness, could be associated with increased emotional distress (Shanahan et al., 2020). Research on previous epidemics involving quarantines has documented declines in psychological health (for a review, see Brooks et al., 2020). Studies documenting distress during the COVID-19 pandemic were rapidly emerging (e.g., Ammar et al., 2020; Wang et al., 2020; Zhang et al., 2020b). During COVID-19 lockdown, higher levels of stress are associated with younger age, being a woman, being single, staying with more children, and living in collectivist cultures (Kowal et al., 2020). Subjectively judged self-control was found to attenuate the link between perceived COVID-19 severity and poorer mental health (Li et al., 2020).

One of the central emotional responses during a pandemic is fear, a negative emotion possibly causing significant levels of mental distress. In spite of the documented worse mental health outcomes, the widespread public fear and anxiety motivated individuals to adopt precautious measures in the early stage of a pandemic (Jørgensen et al., 2020). Individuals who perceived themselves more at risk were found to be likely to comply with protective advice during a pandemic (Brug et al., 2009). Research on disease avoidance demonstrates that people who worry about certain diseases spontaneously remain distant to people outside of their close social circles (Aarøe et al., 2016). Emotionality as a personality trait (characterized by exaggerated levels of anxiety, fear, and emotional reactivity) was associated with a greater level of acceptance of government-mandated personal restrictions (Zettler et al., 2020). In order to balance optimally between strict control measures and their negative impact on psychological responses, it is necessary to identify factors associated with the extent of psychological distress during the pandemic. Specifically, this paper investigated the role of cultural attitudes, social trust, and fear.

## Cultural Orientations

Culture is defined as shared patterns of behaviors and cognitive constructs that shape how people perceive, think about, and act in their social world (Heine, 2010). Different cultures endorse different values regarding individual’s integration with others and the social environment. The most-studied cross-cultural variations are individualism and collectivism (Triandis, 1995; Hofstede, 2011). These values frame individuals’ self-construal as independence vs. interdependence. In individualistic cultures, self-definition is based on individual autonomy and separation from others and priority is given to the individual’s goals and preferences. In collectivistic cultures, the self is defined primarily based on social embeddedness and interdependence with others comprising their ingroups and the priority is placed on the needs, norms, and goals of one’s group or collective. Such distinction has been referred to as a cultural attribute (e.g., Hofstede, 1980) and as a psychological variable of people (e.g., Markus and Kitayama, 1991; Triandis, 1995; Oyserman et al., 2002).

Originally, individualism–collectivism was described as the opposite poles of one continuum (Hofstede, 1980). However, a number of scholars suggested that individualism and collectivism can actually represent two independent continua (e.g., Markus and Kitayama, 1991; Oyserman, 1993). These dimensions have recently been extended to consider cultures’ different emphasis on equality vs. hierarchy (Triandis and Gelfand, 1998; Oyserman et al., 2002; Shavitt et al., 2011), which yields four cultural orientations, namely, horizontal collectivism (HC), vertical collectivism (VC), horizontal individualism (HI), and vertical individualism (VI). Horizontal collectivism describes the tendency to see oneself as similar to others and to emphasize common goals, interdependence, and sociability. Vertical collectivism involves an emphasis on the loyalty to one’s in-group and adherence to hierarchical relations within one’s group. Horizontal individualism is the tendency to want to be unique and distinct from groups and to see individuals as having equality in worth, dignity, and rights. Vertical individualism involves wanting to become distinguished and acquire status, especially through direct competition with others, and it embraces self-assertion to achieve one’s personal aims.

Previous research suggested cultural values may be related to human pathogens. For examples, Fincher et al. (2008) collected cross-cultural data and found out countries with higher pathogen prevalence had higher collectivism and lower individualism scores. Kim et al. (2016)’s survey data of Americans showed that self-reported collectivism related to increased perceived vulnerability to Ebola and xenophobia. Individual-level collectivism was found to be positively correlated with perceived worries and concerns about COVID-19 infection risk (Germani et al., 2020). Self-reported individualism negatively correlated with the intentions to practice social distancing in a primarily American sample (Biddlestone et al., 2020). An analysis of country-level collectivism across 98 countries revealed that country-level collectivism negatively related to both confirmed case and death rates (Webster et al., 2021). Large-scale studies provide evidence that both country-level and state-level indices of collectivism positively predict mask-wearing (Lu et al., 2021). Kemmelmeier and Jami (2021) argued that mask-wearing must be understood on the basis of cultural frameworks, including individualism–collectivism, tightness–looseness, U.S. honor cultures, and political orientation. According to this previous research, people’s responses to the novel pandemic are influenced by cultural frames at both the country-level and the individual-level.

Collectivism–individualism and power distance might work in tandem in influencing people’s considerations regarding collective actions of COVID-19 preventive measures (Zhu et al., 2020). National-level aggregates of Hofstede’s cultural dimensions showed that China scores much lower in individualism and much higher in power distance (Basabe and Ros, 2005; Hofstede, 2011). People in individualistic cultures may be more apt to prioritize their personal convenience or preference over the collective welfare and therefore less willing to wear masks (Lu et al., 2021). However, cultural individualism is not antithetical to social welfare (Waterman, 1981, 1984). According to Watermen, prosocial behavior for individualists can be achieved by taking into account one’s value commitments and individual responsibilities. Direct support for this idea comes from Kemmelmeier et al. (2006), who showed that individualism positively related to charitable giving and volunteerism. Furthermore, when considering mask-wearing as cultural behavior, Kemmelmeier and Jami (2021) demonstrated that independent self-construal indicated a greater intent to wear masks. Based on the literature, people’s responses to a novel pandemic must be interpreted through the individualism–collectivism cultural frameworks.

Beyond country-level differences, there is heterogeneity in the values of individuals residing within the same country. Even though China is considered a collectivistic country, we can distinguish people within the same nation as more or less individualistic/collectivistic, as well as determine who gives more or less importance to equality or hierarchy in relationships. According to Ralston et al. (2014), individual-level dimensions of collectivism and individualism values make a more significant contribution to explaining variances in ethical behaviors than do values at the societal-level. Therefore, the present study examined the individual-level self-report cultural orientations in Chinese college students and its relation to their attitudes toward health compliance and psychological distress. I anticipated that individuals with higher collectivistic orientation would have more favorable evaluations of health compliance as collectivism implies higher tendency to do what is in the best interest of the community. Concerning individuals with higher individualistic orientation, it is not necessarily expected that they disfavor the health compliance as individualism has the potential of promoting prosocial behaviors through self-actualization, individual achievement, and personal autonomy.

In terms of psychological responses during the pandemic, this paper argues that psychological distress may be related to cultural factors. Higher collectivism values more on interdependence and family connectedness (e.g., Heu et al., 2019). The sense of belongingness and social connection may serve as a buffer against psychological distress (Seppala et al., 2013; Yu et al., 2020). Therefore, it was hypothesized that higher collectivistic orientation may be related to less psychological maladjustment in terms of anxiety, stress, and emotional difficulties. Individualism champions the role of individual choice, personal freedom, and self-actualization (Waterman, 1981; Oyserman et al., 2002), which was undermined to some extent during the pandemic lockdown. The more people receive support for their basic psychological needs, the better is their well-being and the better their functioning will be (e.g., De Leersnyder et al., 2015). Hence, higher individualist orientation was hypothesized to be associated with greater psychological distress.

## Trust

Citizen trust in government institutions and unknown others (Uslaner, 2018) is critical in crisis management. Trust in government’s good intentions and capacity to act will foster compliance with regulations as recommended by health authorities (Taylor et al., 2009; van der Weerd et al., 2011; Siegrist and Zingg, 2014). During a pandemic, health officials often give advice on dealing with the virus. As some of the measures and recommendations can be difficult to enforce, trust in the authorities making impartial and beneficial decisions for the society is an important factor to engage in protective behaviors. Past research has found that adolescents embraced social responsibility as a value to live by if they believed that their country is a fair society (Wray-Lake et al., 2016). Social trust was associated with less hoarding and more social distancing behaviors among US adolescents during COVID-19 (Oosterhoff and Palmer, 2020). Trust in the government was also associated with stronger compliance and intentions to report the infection to the authorities (Travaglino and Moon, 2021).

Trust in that fellow citizens will act responsibly facilitates solving problems with collective action, such as vaccination and hoarding of groceries (Lunn et al., 2020). Widespread compliance with coronavirus protective behaviors benefits each individual. This creates the well-known free-rider problem (Olson, 2009), where individuals are in the most comfortable or favorable circumstances if they do not comply themselves while everyone else does. Interpersonal trust may act as a key to buffer against this free-riding problem. Interpersonal trust is defined as believing others in the absence of clear-cut reasons to disbelieve (Rotter, 1980). Previous investigations showed that people who trust more are less likely to lie, cheat, or steal. Likewise, higher interpersonal trust leads to more cooperation and protective behavior changes during a pandemic (Rubin et al., 2009). If people trust their fellow citizens to do the same as they do, they are more likely to contribute to collective action. In contrast, someone with low level of trust might be concerned that others are taking the advantage of someone else practicing social distancing and therefore not follow the distancing instructions.

## Worry of Covid-19

Research in health psychology has long recognized that disease-specific worry motivates preventative health behaviors (see in Sweeny and Dooley, 2017; McCaul et al., 2020). Worry can be meaningfully conceptualized as a flexible resource that may help people bring up issues and motivate proactive behaviors to solve such issues (Bazzoli et al., 2021). Worrying about a stressor (e.g., the novel coronavirus) keeps the stressor and its feared outcomes at the forefront of one’s mind, provides frequent and continuous cues to action, and sustains motivation toward action. Individuals who perceived themselves more at risk were found to be likely to comply with protective advice during a pandemic (Brug et al., 2009). Research on disease avoidance demonstrates that people who worry about certain disease spontaneously remain distant from people outside of their close social circles (Aarøe et al., 2016).

## The Present Study

The rationale of the present research was to evaluate (a) Chinese college students’ attitudes toward health compliance, (b) their psychological responses in terms of distress, anxiety, and depression, and (c) the roles of individual-level cultural orientations, interpersonal trust, and worries and concerns about COVID-19 in predicting attitudes toward compliance and psychological responses. Specifically, this study measured the individual-level of HC, VC, HI, VI cultural orientations in Chinese college students during the COVID-19 pandemic. Data on the participants’ worries and concerns about the virus, interpersonal trust level, psychological distress, and compliance attitude were also collected through questionnaires. Hypotheses were formed as follow given the literature reviewed above:

Hypothesis 1 (H1): Concerning compliance attitude, individuals who have higher tendency of collectivism and individualism are more ready to follow health advice.

Hypothesis 2 (H2): Higher levels of interpersonal trust and greater fear positively predict attitudes toward compliance.

Hypothesis 3 (H3): Concerning psychological responses, individual cultural orientations should correlate with psychological distress.

## Materials and Methods

### Participants

A cross-sectional survey was conducted to investigate the university students’ attitudes toward public health interventions. A total of *N*=384 university students (*M*age=19.3years, *SD*=0.94, 84% female) participated in the anonymous online questionnaire *via* SurveyStar and, in exchange, received credit in a health psychology course. The data collection occurred between April 9 and 16, 2020. Most participants lived in East China during the COVID-19 pandemic.

### Procedures and Materials

Participants initially provided their informed consent before answering to the questionnaire. On average, the questionnaire took 12.69min to complete. This procedure followed Chinese Psychological Society ethical standards and was approved by an institutional ethical review panel prior to data collection. The questionnaire consisted of questions that covered several areas: (1) demographic data; (2) attitudes toward compliance; (3) worries and concerns about COVID-19; (4) mental health status; (5) cultural orientations; and (6) interpersonal trust.

#### Demographics

Participants were asked to report their gender, age, education, current residential region, quarantine status, and contact history with someone with confirmed or suspected COVID-19.

#### Attitudes Toward Compliance

Respondents were asked to report the degree of their willingness to accept and follow the protective measures as suggested by the authorities. The protective measures against COVID-19 included wearing a mask, personal hygiene, limited social contact, school closure and online course, disapproval of family gathering, connecting with friends *via* digital, avoiding travel, avoiding crowded place, staying home, prohibiting visiting, avoiding eating in restaurants, avoiding public transportation, covering mouth when coughing and sneezing, receiving temperature check anywhere and anytime, and community lockdown when confirmed cases were found. Participants reported their attitudes toward compliance on a 5-point scale from “1-Absolutely not willing to” to “5-Totally willing to.” There were 20 items in total, *Cronbach’s α*=0.90.

#### Worries and Concerns About COVID-19

The scale consisted of 13 items (e.g., *I am fearful of being infected*, *I am worried that my family will be infected*) measuring one’s worries and concerns about COVID-19. Participants are asked to rate their agreement with each statement on a 6-point scale from “1-Strongly Disagree” to “6-Strongly Agree” *Cronbach’s α*=0.88.

#### Mental Health Status

The psychological distress of COVID-19 was measured using the Impact of Event Scale-Revised (IES-R, Creamer et al., 2003), Generalized Anxiety Disorder (GAD-7, Spitzer et al., 2006), and Self-Rating Depression Scale (SDS, Thurber et al., 2002). The IES-R has been well-validated in the Chinese population for determining the extent of psychological distress after exposure to a public health crisis. This 22-item questionnaire measured avoidance, intrusion, and hyperarousal, specifically in response to the event of COVID-19 outbreak (Wang et al., 2020). Participants rated each item on a five-point scale from “1-Never” to “5-Always.” The GAD-7 and SDS has been demonstrated to be a reliable and valid measure in assessing mental health in the Chinese population. GAD-7 measured state anxiety, and SDS measured depression on four-point Likert scale from “1-Occasionally” to “4-Frequently.” There were 4 reverse items in the SDS scales. Internal consistencies for IES, GAD-7, and SDS were 0.95, 0.93, and 0.84, respectively.

#### Cultural Orientation

Based on Triandis (1995), individualism and collectivism can be measured as personality constructs at the individual-level. The original individualism and collectivism scale (ICS, Singelis et al., 1995) is a 32-item scale, and 8 items each are used to measure HI (e.g., *I enjoy being unique and different from others in many ways*), VI (e.g., *Competition is the law of nature*), HC (e.g., *If a coworker gets a prize, I would feel proud*), and VC (e.g., *I usually sacrifice my self-interest for the benefits of my group*). In this study, the 28-item version of the original scale was applied as this modified version has been validated with Chinese participants (Huang et al., 2013). Each statement was rated on a seven-point scale from “1-Strongly disagree” to “7-Strongly agree.” The reliabilities for the dimensions of HI, VI, HC, and VC were 0.78, 0.87, 0.82, and 0.76.

#### Trust

Rotter’s Interpersonal Trust Scale was used to measure one’s expectation that the behavior, promises, or statements of other individuals can be relied upon (Rotter, 1967; Chun and Campbell, 1974). There were 24 items in this scale (e.g., *Most people can be counted on to do what they say they will do*) and half of them are reverse coded (e.g., *Even though we have reported in newspapers, radio, and television, it is hard to get objective accounts of public events*). The response format was a five-point Likert scale (“1-Strongly disagree” to “5-Strongly agree”). The questionnaire has been translated into Chinese version and validated in the Chinese population, *Cronbach’s α*=0.71.

### Data Analysis

Descriptive statistics were carried out. Correlations between measures of attitudes toward compliance, psychological responses including distress, anxiety, and depression, cultural orientations at the individual-level, interpersonal trust, and worries and concerns about COVID-19 were carried out using Pearson and Spearman rank correlations. A composite score (CS) of psychological responses was calculated as the mean of the standardized scores of the IES Score, GAD-7, and DSS, which were significantly and positively correlated to each other with a large effect size. The reliability of all items comprising the CS in assessing psychological responses was 0.95. The attitudes toward compliance and CS were the outcome variables of two multiple regression models.

## Results

Overall, none of the participants had been forced into quarantine, neither been in contact with an individual with suspected or confirmed COVID-19. Only 0.8% reported indirect contact with the confirmed cases. As shown in Table 1, overall participants reported high mean score of the attitudes toward compliance and low mean score of the psychological response measures. The average scores of HI, HC, and VC were higher than VI.

**Table 1:** Descriptive statistics for key variables (*n*=384).

Psychological variables	Possible range	*M±SD*	Skewness	Kurtosis
Attitudes toward compliance	1–5	4.48±0.48	−1.04	0.56
**Psychological responses**				
Distress (IES)	1–5	1.92±0.67	1.37	3.41
Anxiety (GAD-7)	1–4	1.58±0.61	1.38	2.21
Depression (SDS)	1–4	1.67±0.49	1.04	1.13
**INDCOL**				
HI	1–7	4.79±0.95	−0.45	1.68
VI	1–7	3.99±1.11	0.01	0.40
HC	1–7	5.01±0.87	−0.45	1.91
VC	1–7	4.87±0.76	0.20	1.53
**Other predictors**				
Interpersonal Trust	1–5	2.90±0.32	−0.54	2.44
Worries and concerns about the COVID-19	1–6	2.93±0.96	−0.01	−0.25

*IES, Impact Event Scale; GAD-7, Generalized Anxiety Disorder; SDS, Self-Rating Depression; INDCOL, Horizontal and Vertical Individualism and Collectivism Scale; HI, horizontal individualism; VI, vertical individualism; HC, horizontal collectivism; VC, vertical collectivism*.

Table 2 shows both parametric and non-parametric correlations among all the variables. As shown in Table 2, HI, HC, and VC were significantly and positively related to attitudes toward compliance. The measures of psychological responses in terms of distress, anxiety, and depression were highly correlated. It is important to note that HI, VI, and VC were positively associated with the psychological response measures, while interpersonal trust negatively correlated with anxiety, depression, and worries and concerns about COVID-19.

**Table 2:** Correlations between the key variables (*n*=384).

	1	2	3	4	5	6	7	8	9	10	11
1. Gender	/	0.09	−0.05	0.03	0.05	−0.05	0.03	−0.02	0.01	−0.02	−0.03
2. Attitudes toward compliance	0.13^*^	/	0.01	−0.06	−0.09	0.04	0.13^*^	−0.09	0.15^*^	0.15^*^	0.07
3. Distress	−0.06	0.02	/	0.50^**^	0.33^**^	0.37^**^	0.03	0.28^**^	0.00	0.08	−0.09
4. Anxiety	−0.01	−0.08	0.47^**^	/	0.60^**^	0.21^**^	0.07	0.29^**^	−0.05	0.07	−0.19^**^
5. Depression	0.02	−0.10	0.37^**^	0.71^**^	/	0.18^*^	0.15^*^	0.27^**^	0.03	0.07	−0.22^**^
6. Worries and concerns	−0.04	0.04	0.39^**^	0.24^**^	0.22^**^	/	0.05	0.12^*^	0.03	0.09	−0.10
7. HI	0.05	0.15^*^	0.12^*^	0.13^*^	0.24^**^	0.08	/	0.34^**^	0.16^*^	0.22^**^	−0.15^*^
8. VI	−0.01	−0.07	0.35^**^	0.35^**^	0.35^**^	0.14^*^	0.44^**^	/	0.00	0.13^*^	−0.26^**^
9. HC	0.04	0.20^**^	0.11^*^	0.00	0.09	0.04	0.26^**^	0.07	/	0.59^**^	0.17^*^
10. VC	0.00	0.19^**^	0.20^**^	0.14^*^	0.15^*^	0.12^*^	0.26^**^	0.20^**^	0.63^**^	/	0.02
11. Trust	−0.02	0.11^*^	−0.06	−0.19^**^	−0.24^**^	−0.11^*^	−0.16^*^	−0.28^**^	0.22^**^	0.08	/

*Above the diagonal is Spearman rank correlation coefficient and below the diagonal is Pearson correlation coefficient*.

* *p< 0.05*;

** *p< 0.001*.

The attitudes toward compliance and composite score of psychological responses were the outcome variables of two multiple regression models. The predictors included gender, cultural orientations, trust, worries, and concerns about COVID-19. As shown in Table 3, both multiple regression models were significant. The model predicting attitudes toward compliance was significant with small effect size [*F*(7, 376)=5.91, *p*<0.001, *f*^2^=0.11] and explained 9.9% of the variance of attitudes toward compliance (*R*^2^=0.099). Controlling for the effect of gender, HI and VC significantly and positively predicted the attitudes toward compliance, while VI negatively predicted attitudes toward compliance. In the regression model to predict the composite score of psychological responses, the results showed that the model was significant [*F*(7, 376)=40.60, *p*<0.001, *f*^2^=0.76] and explained 43.1% of the variance of psychological responses (*R*^2^=0.431). Controlling for the effect of worries and concerns of COVID-19, VI and VC still significantly predicted more negative psychological responses but HC significantly predicted less psychological distress. Table 4 revealed the three separate regression model with psychological distress, anxiety, and depression as the dependent variables. The results were similar to that for the composite score model except for higher trust, which significantly predicted lower levels of depression.

**Table 3:** Multiple regression models of gender, cultural orientations, trust on attitudes toward compliance, and negative psychological responses.

Predictor	Attitudes toward compliance		Negative psychological responses			
	*B (95% CI)*	*t*	*p*	*B (95% CI)*	*t*	*p*
Gender	0.15(0.03, 0.28)	2.36	0.019	−0.02(−0.12, 0.08)	−0.41	0.679
HI	0.09(0.03, 0.14)	2.98	0.003	−0.02(−0.07, 0.02)	−0.99	0.323
VI	−0.06(−0.11, −0.01)	−2.55	0.011	0.13(0.09, 0.17)	6.49	<0.001
HC	0.04(−0.03, 0.11)	1.19	0.236	−0.11(−0.17, −0.05)	−3.70	<0.001
VC	0.08 (0.00, 0.16)	1.98	0.049	0.09(0.03, 0.16)	2.86	0.005
Trust	0.10(−0.06, 0.26)	1.21	0.229	−0.08(−0.21, 0.04)	−1.31	0.191
Worries and concerns about COVID-19	−0.03(−0.08, 0.02)	−1.07	0.284	0.25(0.21, 0.29)	11.92	<0.001
	*F*(7, 376)=5.91, *p* <0.001 *R*^2^ =0.099			*F*(7, 376)=40.6, *p* <0.001 *R*^2^ =0.431		

*Gender was coded as 1=male, 2=female*.

**Table 4:** Multiple regression models of gender, cultural orientations, trust on respective psychological responses of distress, anxiety, and depression.

Predictor	Distress		Anxiety				Depression		
	*B*	*t*	*p*	*B*	*t*	*p*	*B*	*t*	*p*
Gender	−0.08	−1.07	0.287	0.01	0.11	0.910	0.01	0.15	0.882
HI	−0.04	−1.28	0.202	−0.03	−0.84	0.401	0.00	0.09	0.929
VI	0.15	5.25	<0.001	0.13	4.73	<0.001	0.10	4.55	<0.001
HC	−0.06	−1.42	0.158	−0.12	−2.79	0.006	−0.15	−4.59	<0.001
VC	0.10	2.05	0.041	0.11	2.41	0.016	0.07	1.93	0.054
Trust	0.15	1.62	0.106	−0.09	−1.01	0.312	−0.31	−4.41	<0.001
Worries and concerns about COVID-19	0.35	11.34	<0.001	0.23	7.99	<0.001	0.16	7.03	<0.001
	*F*(7, 376)=30.45, *p* <0.001, *R*^2^ =0.362			*F*(7, 376)=19.77, *p* <0.001 *R*^2^ =0.269			*F*(7, 376)=25.29, *p* <0.001 *R*^2^ =0.320		

## Discussion

In order to implement measures against the spread of virus, it is important to understand what factors predict individual’s attitude to comply with the advice from health authorities. The goal of this study was to examine the attitudes toward health compliance and the psychological responses within the context of individualism–collectivism cultural frameworks. Data from Chinese university students suggested that individual differences in cultural orientations were significantly predictors of attitudes toward compliance and psychological responses.

The current study evaluated Chinese participants’ attitudes toward compliance during the pandemic. The results showed a high mean score of attitudes toward compliance, suggesting that the restrictive measures were generally endorsed by Chinese participants. In a recent cross-cultural research on individuals’ evaluation of COVID-19 preventive measures, Chinese participants indicated the highest acceptance of society-level preventative measures, but not individual-level preventative measures, compared to their Japanese and US counterparts (Zhu et al., 2021). Because the Chinese government used strict isolation measures to combat the spread of COVID-19, the majority of Chinese citizens cooperated to practice social distancing behaviorally. From a social domain perspective, people in collectivistic societies also aspired to make personal choices and have freedoms, and rights, which sometimes contradicted the dominant cultural tenets that, for instance, emphasized societal welfare (Turiel, 2002). Although Chinese participants behaviorally followed the restrictive measures enforced by the police and government, mentally they might have personal considerations and lower their acceptance of individual-level precautions (e.g., wearing gloves when shopping and self-disclosing of traveling history).

To understand better what might contribute to Chinese participants’ attitudes toward compliance, the current study examined several effective predictors. One of them was gender. Results indicated that female Chinese students were more willing to comply than males. Other than gender, individual-level cultural orientations predicted the participants’ willingness to comply in different ways. Partly consistent with the hypothesis, VC, but not HC, predicted positive attitudes toward compliance. The COVID-19 pandemic required rapid public compliance with advice from health authorities and collective actions, while following the advice might cause inconvenience in personal life. HC orientation fostered in-group commitment with individuals forming a strong sense of shared social identity. But seeing the self as extremely similar and equal to each other was not enough to elicit strong acceptance of the preventive measures. When conflicts between collective and individual interests existed, it required ones to sacrifice their own interests to the collective benefits. Higher VC implied a stronger group identity, social disapproval for those who did not comply, and more respects for authority. Hence, VC promoted the intentions to adopt prevention behaviors better than HC did. This result was consistent with the country-level analyses (Lu et al., 2021; Webster et al., 2021). The reasons for East Asia’s effective control of the pandemic were suggested to be civic responsibility (including heightened levels of concern for the health of others over personal freedom and convenience) underpinned by collectivist norms (including the public’s willingness to call individuals out for failing to comply with safety rules; Liu et al., 2020). To explain why people cooperated rather than competed in response to a crisis, factors included an emerging sense of shared identity and concern for others (Van Bavel et al., 2020). This speculation was supported with the evidence from Western participants that framing prevention behaviors as benefiting others was more effective than framing them as beneficial for oneself (Jordan et al., 2020).

It was noteworthy that the HI orientation significantly predicted attitudes toward compliance. These results suggested that individuals who strived to be distinct without desiring special status were more likely to agree to comply with health advice. The horizontal view of individualism emphasizes independence and equality among members where egalitarian norms are observed. Autonomy and independent self-construal possibly facilitated the willingness to take personal responsibility to follow the physical restrictive measures (Waterman, 1981, 1984). This is consistent with Kemmelmeier and Jami (2021)’s finding that independent self-construals emerged as an important predictor of mask-wearing behaviors. Although individuals high in independence resented masks more than their low-independence counterparts, they assumed the personal responsibility to carry out the mask-wearing behavior because it was beneficial.

In contrast, VI orientation predicted less favorable attitudes toward compliance. Individualists seeing themselves different from others in terms of status inequality might impede compliance with health advice. These results imply that individualism was not the major obstacle to practicing social distancing. The extent to which individualists valued competition and inequality distinguished their attitude to follow the health advice. Individuals with high VI orientation emphasized competition and uniqueness, which might reduce their willingness to cooperate with the strict behavioral measures. On this basis, authorities could still potentially foster further compliance by appealing to personal responsibility and self-sacrifice in individualist cultures.

Contrary to prior research on epidemics (Jørgensen et al., 2020; Zickfeld et al., 2020), the current data revealed that worries and concerns of the virus were not an effective predictor of the positive attitudes toward compliance in Chinese cultural context. From previous epidemic studies, risk perceptions and fear were major and culturally consistent factors related to behavioral changes to adopt the health recommendations (Tannenbaum et al., 2015). When deciding whether to engage with proposed health solutions, people consider their susceptibility to the threat and its severity. Risks are judged to be greater when they have more emotional impact. Therefore, fear tends to increase perception of risk, which leads to persuasion regarding to attitude and behaviors. Research from early cases of COVID-19 found that the intentions to implement behavioral changes were most strongly predicted by risk perception (Brouard et al., 2020; Harper et al., 2020). In contrary to this evidence, the present study on Chinese university students found that worries and concerns about the disease were not enough to elicit willingness to self-isolate. On the other hand, the increased sense of worries and concerns entailed mental health costs for the public (Ornell et al., 2020). Our results also showed that the measures of worries and concerns about COVID-19 were highly associated to the measures of negative psychological responses.

Given the complexity and level of uncertainty regarding the risks, dangers, and future outcomes of the COVID-19 pandemic, the importance of psychological adjustment was paramount. From the regression model, psychological distress was positively predicted by VI and VC and negatively predicted by HC, when the effect of worries and concerns of COVID-19 was controlled. Same as Italian emerging adults (Germani et al., 2020), Chinese university students who had a stronger will to distinguish themselves from others and acquire status through individual competitions with others demonstrated higher degree of negative psychological impact. It suggested that young adults were finding more difficulties and struggling to make future plans during the pandemic period characterized by instability especially when they could not take advantage by excising their competition mindset. Consequently, individualistic people thinking of the self as unique and competitive experienced more negative psychological effects because of a lack of feeling connection with others. Collectivistic people who prioritized in-group goals over personal goals were more likely to experience negative psychological responses because they were used to suppress their own needs and feelings, which endangered the mental health and well-being. The current result showed that HC significantly predicted less psychological problems. People who were horizontal collectivists cooperated with their in-group and emphasized equal responsibility shared by their group member. Such cultural disposition could possibly result in firm belief that fellow citizens were engaging in protective behavior and they were not personally threatened. Therefore, less psychological maladjustment was experienced.

Although trust was correlated with the attitudes toward compliance and psychological distress, it did not significantly predict the two dependent variables in the regression models. These results indicated that individual-level of cultural orientations was more effective in accounting for the attitude of compliance and psychological responses than interpersonal trust. In the model predicting depression, social trust was negatively associated with major depression. Higher levels of social trust might provide individuals with social support and other resources that might reduce the effects of stressors on mental health. Furthermore, high social trust might facilitate health-promoting behaviors and social connections with others, leading to lower rates of depression. This result suggested that social trust might serve as a buffer and help people cope more effectively with the difficulties during the pandemic.

Before concluding, there are some caveats that need to be addressed in future studies. First, the generalizability of the findings was limited to Chinese university students (mainly females). It was rather homogeneous sample and not representative of the whole country or other age groups. With the small effect size discovered in the current study, one should be cautious about generalizing the conclusions to other populations. College students might also have unique characteristics relevant to their acceptance of COVID-19 prevention effects. For example, their educational background facilitated a trust in science, which increased their endorsement of the preventive measures. At the same time, college students also belonged to low-fatality age groups for COVID-19, which might lead to underestimation of their risks and less acceptance of extreme restrictive measures. Further research is needed to expand the findings of this study to a broader age range with more diverse backgrounds.

Moreover, the current study only examined the individual-level of cultural values within the same cultural background. It cannot be determined with certainty whether the results can be generalized to other national contexts. The cultural environment might affect the expression of individual-level cultural disposition. Since this study only examined participants from one specific culture, a clear direction of causality between the selected variables should be tested and established in further cross-cultural research. Other than the cultural dimensions of individualism–collectivism, future research, including individuals from a larger number of societies, should take pandemic preparedness and severity into consideration, as well as other society-level characteristics (e.g., economic development, public health infrastructure, political stability) that may contribute to cross-society differences in attitudes toward preventive measures. For example, the community-level tendency to engage with strangers and freely choose friends, called relational mobility, robustly predicted the growth curves of confirmed cases of and deaths due to COVID-19 (Salvador et al., 2020).

To conclude, the efforts to combat COVID-19 depend not only on how many resources a government can muster or how stringent their policies are, but also on the support and cooperation of citizens with the preventive measures. To complement the knowledge about individuals’ attitudes toward the health compliance, the present study showed that Chinese college students were more willing to comply with social distancing and other protective behaviors if they had higher vertical collectivist and horizontal individualistic cultural orientations. Moreover, psychological distress was less likely to occur in individuals with higher horizontal collectivist tendency. These findings also have important implications for public health policymakers in different societies who seek to stimulate public cooperation with preventive measures. For example, appealing to people’s sense of societal integrity and collective welfare might be as effective as emphasizing a sense of control and personal responsibility. For messaging and interventions to be effective, it is vital to incorporate the types of beliefs and concerns that individuals in different contexts endorse. Hence, it is possible to come up with effective strategies to fight against infectious disease without compromising the core values of democracy.

## Data Availability Statement

The raw data supporting the conclusions of this article will be made available by the authors, without undue reservation.

## Ethics Statement

The studies involving human participants were reviewed and approved by University ethics committee. The patients/participants provided their written informed consent to participate in this study.

## Author Contributions

WX contributed to the design and implementation of the research, to the analysis of the results, and to the writing of the manuscript.

## Funding

This project was funded in part by The National Social Science Fund of China, 19CYY001.

## Conflict of Interest

The author declares that the research was conducted in the absence of any commercial or financial relationships that could be construed as a potential conflict of interest.

## Publisher’s Note

All claims expressed in this article are solely those of the authors and do not necessarily represent those of their affiliated organizations, or those of the publisher, the editors and the reviewers. Any product that may be evaluated in this article, or claim that may be made by its manufacturer, is not guaranteed or endorsed by the publisher.
